# Synthesis and Biological Evaluation of 2-Substituted Benzyl-/Phenylethylamino-4-amino-5-aroylthiazoles as Apoptosis-Inducing Anticancer Agents

**DOI:** 10.3390/molecules25092177

**Published:** 2020-05-06

**Authors:** Paola Oliva, Valentina Onnis, Elisa Balboni, Ernest Hamel, Francisco Estévez-Sarmiento, José Quintana, Francisco Estévez, Andrea Brancale, Salvatore Ferla, Stefano Manfredini, Romeo Romagnoli

**Affiliations:** 1 Dipartimento di Scienze Chimiche e Farmaceutiche, University of Ferrara, Via L. Borsari 46, 44121 Ferrara, Italy; lvopla@unife.it (P.O.); elisa02.balboni@student.unife.it (E.B.); rmr@unife.it (R.R.); 2 Dipartimento di Scienze della Vita e dell’Ambiente, University of Cagliari, University Campus, 09042 Monserrato (Cagliari), Italy; 3 Screening Technologies Branch, Developmental Therapeutics Program, Division of Cancer Treatment and Diagnosis, Frederick National Laboratory for Cancer Research, National Cancer Institute, National Institute of Health, Frederick, MD 21702, USA; hamele@dc37a.nci.nih.gov; 4 Departamento de Bioquímica y Biología Molecular, Instituto Universitario de Investigaciones Biomédicas y Sanitarias, Universidad de las Palmas de Gran Canaria, E-35016 Las Palmas de Gran Canaria, Spain; festevez1985@gmail.com (F.E.-S.); jose.quintana@ulpgc.es (J.Q.); francisco.estevez@ulpgc.es (F.E.); 5 School of Pharmacy and Pharmaceutical Sciences, Cardiff University, King Edward VII Avenue, Cardiff CF10 3NB, UK; brancalea@cardiff.ac.uk (A.B.); ferlas1@cardiff.ac.uk (S.F.); 6 Dipartimento di Scienze della Vita e Biotecnologie, Università di Ferrara, 44121 Ferrara, Italy; stefano.manfredini@unife.it

**Keywords:** microtubules, structure-activity relationship, antiproliferative activity, pharmacophoric merging, apoptosis

## Abstract

Induction of apoptosis is a common chemotherapeutic mechanism to kill cancer cells The thiazole system has been reported over the past decades as a building block for the preparation of anticancer agents. A novel series of 2-arylalkylamino-4-amino-5-(3′,4′,5′-trimethoxybenzoyl)-thiazole derivatives designed as dual inhibitors of tubulin and cyclin-dependent kinases (CDKs) were synthesized and evaluated for their antiproliferative activity in vitro against two cancer cell lines and, for selected highly active compounds, for interactions with tubulin and cyclin-dependent kinases and for cell cycle and apoptosis effects. Structure-activity relationships were elucidated for various substituents at the 2-position of the thiazole skeleton. Among the synthesized compounds, the most active analogues were found to be the *p*-chlorobenzylamino derivative **8e** as well as the *p*-chloro and *p*-methoxyphenethylamino analogues **8f** and **8k**, respectively, which inhibited the growth of U-937 and SK-MEL-1 cancer cell lines with IC_50_ values ranging from 5.7 to 12.2 μM. On U-937 cells, the tested compounds **8f** and **8k** induced apoptosis in a time and concentration dependent manner. These two latter molecules did not affect tubulin polymerization (IC_50_ > 20 μM) nor CDK activity at a single concentration of 10 μM, suggesting alternative targets than tubulin and CDK for the compounds.

## 1. Introduction

Apoptosis, a form of programmed cell death, plays a critical role in normal cell development and is a highly controlled mechanism that regulates the selective removal of damaged and dysfunctional cells [1]. This process was recognized as an important phenomenon for successful cancer chemotherapy [2], and identification of compounds that can activate apoptosis in cancer cells is recognized as one of the most promising approaches for the discovery and development of potential anticancer agents [3,4]. Recently, several efforts were made for the discovery of new small molecules that target both tubulin and cyclin-dependent kinases (CDKs), which are involved in regulating the cell cycle, promoting tumor cell proliferation and apoptosis [5,6].

Microtubule-targeting agents act as inhibitors in the mitotic phase because they disrupt the G_2_-M transition, thus inducing cell-cycle arrest and apoptosis in tumor cells [7,8]. Antimitotic agents that inhibit microtubule function are one of the major classes of cytotoxic drugs for cancer treatment and represent a validated approach for designing anticancer agents in a clinical setting, including the taxanes (paclitaxel and docetaxel) and the vinca alkaloids (vinblastine and vincristine) [9].

**Figure 1:**
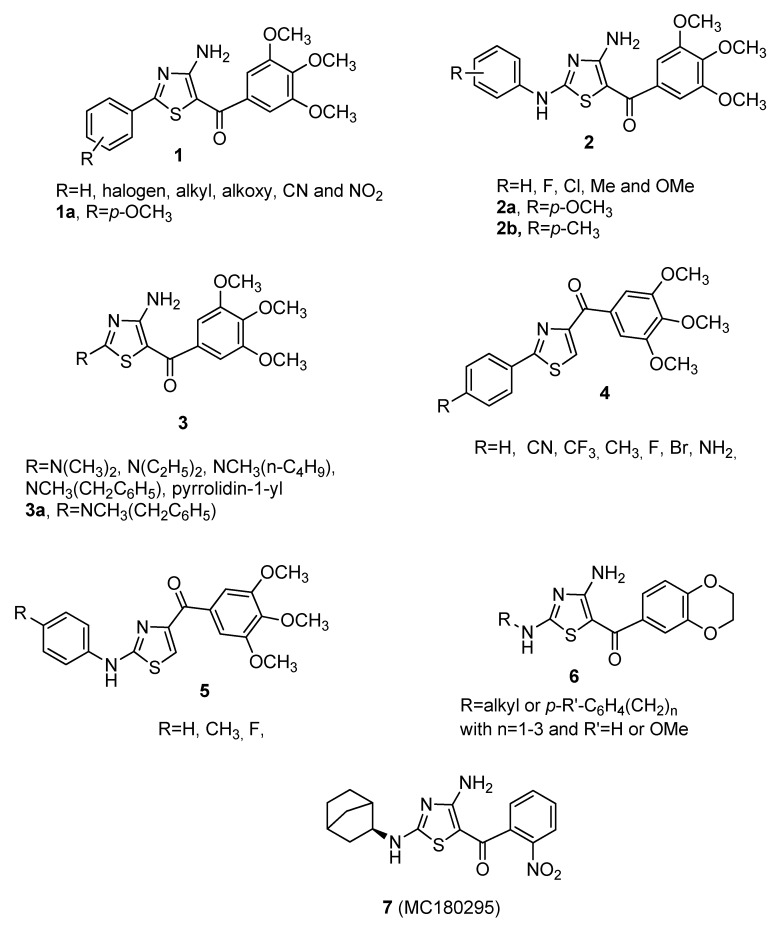
Chemical structures of 2-substituted aroylthiazole derivatives **1**–**7**.

Among the inhibitors of tubulin polymerization characterized by the presence of a thiazole ring, there have been several reports describing structurally simple synthetic molecules based on the 2-substituted-4/5-(3,4,5-trimethoxybenzoyl)thiazole skeleton [10,11,12,13,14]. We have described different series of 2-substituted-4-amino-5-(3′,4′,5′-trimethoxybenzoyl)thiazoles that show strong antiproliferative activity against tumor cell lines, and these compounds inhibit tubulin polymerization by interfering with the colchicine site. As a consequence, the cells arrest in the G_2_-M phase of the cell cycle [10,11,12]. The structural requirements that elicit potent activity were aryl, arylamino and various cyclic or acyclic alkylamino (in particular pyrrolidine) substituents at the 2-position of 4-aminothiazole nucleus, to furnish compounds with general structures **1**, **2** and **3**, respectively. In structure-activity relationship studies, we examined the effects of electron-withdrawing groups (EWGs) or electron-releasing groups (ERGs) at the phenyl moiety (Figure 1). Miller and co-workers also discovered two novel classes of simple synthetic molecules based on a 4-(3,4,5-trimethoxybenzoyl)thiazole molecular skeleton, characterized by the presence of an aryl or an anilino moiety at the 2-position of the thiazole ring and corresponding to conjugates with general structures **4** and **5**, respectively. These conjugates showed nanomolar antiproliferative activity against cancer cell lines by inhibiting tubulin polymerization [13,14].

Cyclin-dependent kinases (CDKs) are serine/threonine protein kinases that play pivotal roles in the regulation of numerous important molecular and cellular processes [15]. CDK1, 2, 4 and 6 play a crucial role in cell cycle progression, while CDK7, 8 and 9 are involved in controlling gene transcription [16]. CDKs have long been considered promising therapeutic targets for the treatment of diseases, including cancer [17,18]. The recent therapeutic success of two dual CDK4/6 inhibitors, palbociclib [19] and ribociclib [20], approved by the FDA for the treatment of patients with estrogen receptor (ER)-positive and HER2-negative advanced breast cancer, respectively, has renewed interest in the therapeutic potential of small-molecule inhibitors for CDKs.

A Temple University group has reported identification by high-throughput screening (HTS) of novel 2-aryl/alkylamino-4-amino-5-aroylthiazoles as CDK inhibitors, selected from a series with general structure **6** [21]. The compounds of the invention appeared to be more potent than other CDK inhibitors, which had not been developed by targeting silenced tumor-suppressor gene expression. Issa and co-workers also identified the 2-(*exo*-2′-aminonorbornane)-4-amino-5-(2′-nitrobenzoyl)-thiazole derivative **7** (MC180295) as being active at low nanomolar levels against CDK9, and compound **7** was at least 22-fold more selective for CDK9 than for other CDKs [22].

Single-target cancer chemotherapeutic agents have several limitations, including limited efficacy, drug resistance, significant adverse effects, and toxicities [23]. As a result, to overcome these drawbacks, multitargeting antitumor agents has emerged as an effective strategy in drug discovery research [24]. Multitargeting agents comprise the incorporation of two or more pharmacophores of bioactive scaffolds in one molecule and are designed to simultaneously address more than one biological target. The goal is to achieve a synergistic effect [25,26].

**Figure 2:**
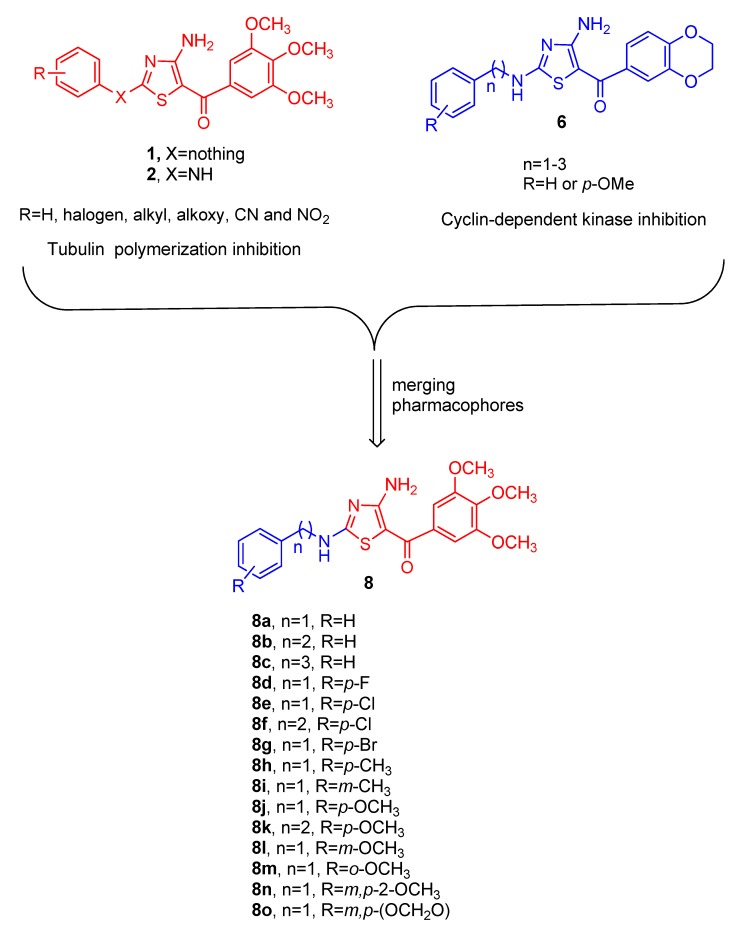
Design strategy for the preparation of 2-alkylamino-4-amino-5-(3′,4′,5′-trimethoxy-benzoyl)thiazoles **8a**–**o**.

Based on these considerations, the current study was designed to synthesize a new series of potential dual tubulin and CDK inhibitors with general structure **8** by a pharmacophore fusion approach (Figure 2). These molecules were characterized by the presence of a common 4-amino-5-(3′,4′,5′-trimethoxybenzoyl)thiazole scaffold, which was identified as the minimum structural requirement for the optimal antitubulin activity of compounds with general formulae **1**–**3**, and by performing modifications at the 2-position of the thiazole ring by replacing the aryl, anilino or alkylamino side chains with a benzylamino-/phenylethylamino moiety, which was beneficial for the CDK inhibitory activity of compound **6**. For the newly synthesized compounds, modifications were focused on varying the length of the linker (from one to three methylene units) between the nitrogen at the 2-position of the thiazole ring and the phenyl ring, and this latter moiety was substituted with electron-releasing (CH_3_ or OCH_3_) or electron-withdrawing (F, Cl or Br) groups. All the newly synthesized agents contained the 3′,4′,5′-trimethoxyphenyl group in the benzoyl moiety at the C-5 position of the thiazole ring, a well-known characteristic structural requirement that is in a large series of inhibitors of tubulin assembly, such as colchicine, combretastatin A-4 (CA-4) and podophyllotoxin [27], and usually maximizes activity.

## 2. Chemistry

**Scheme 1:**
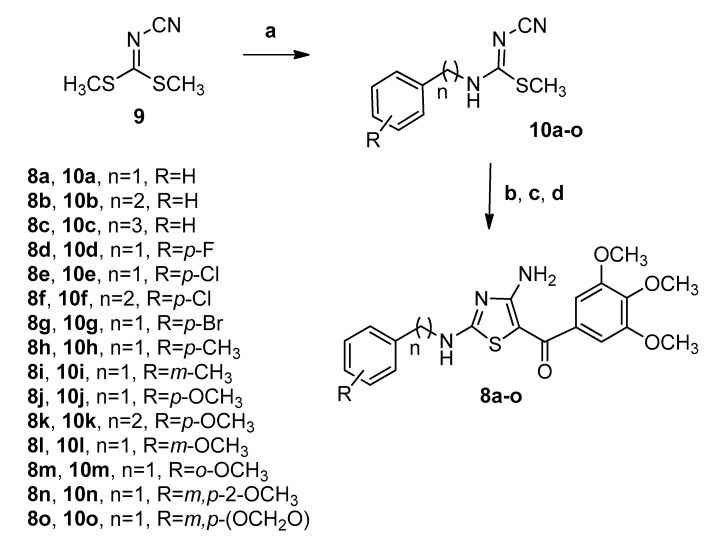
Reagents (**a**) Appropriate ArCH_2_NH_2_ or Ar(CH_2_)_2_NH_2_ or C_6_H_5_(CH_2_)_3_NH_2_, i-PrOH, rt; (**b**) Na_2_S, DMF, 80 °C, 1.5 h; (**c**) 1-(3′,4′,5′-trimethoxyphenyl)-2-bromoethanone, 50 °C, 2 h; (**d**) K_2_CO_3_, 1 h.

Synthesis of compounds **8a-o** was accomplished using the procedure described in Scheme 1. The condensation of dimethyl cyanodithioimidocarbonate **9** with the appropriate benzyl-/ phenylethylamine or 3-phenylpropylamine resulted in the formation of imidates **10a**–**o**, which were cyclized into the corresponding 2-substituted-4-amino-5-(3′,4′,5′-trimethoxybenzoyl)thiazoles **8a**–**o** by a one-pot three-step sequential procedure starting from **10a**–**o**, which was reacted with sodium sulfide, followed by the addition of 1-(3′,4′,5′-trimethoxyphenyl)-2-bromoethanone and finally cyclized with potassium carbonate.

## 3. Results and Discussion

### 3.1. In Vitro Antiproliferative Activities against Human U-937 and SK-MEL-1 Cell Lines

In Table 1, we summarize the antiproliferative activity of the 15 novel 2-arylalkylamino-4-amino-5-(3′,4′,5′-trimethoxybenzoyl)thiazole derivatives **8a**–**o** against human leukemia (U-937) and melanoma (SK-MEL-1) tumor cells lines. Cells were cultured for 72 h, and the IC_50_ values were calculated using a colorimetric MTT assay. These data were compared with those of the corresponding previously published 2-*p*-methoxyphenyl and 2-*p*-methoxyanilino derivatives **1a** and **2a**, respectively. Doxorubicin and etoposide, which are among the most effective anticancer agents, were used as positive controls, along with CA-4 as a well-known inhibitor of tubulin polymerization.

Two compounds, **8e** (*p*-chlorobenzylamino) and **8k** (*p*-methoxyphenylethylamino), have the best antiproliferative activities against these cancer cell lines, with single-digit micromolar IC_50_ values, and, overall, were more active than the *p*-methoxyphenyl derivative **1a** [22]. Along with these molecules, only the *p*-chlorophenethylamino analogue **8f** had a low micromolar IC_50_ value against U-937 cells. Starting from the previously published *p*-methoxyanilino derivative **2a**, the insertion of a methylene or ethylene spacer between the nitrogen and *p*-methoxyphenyl ring, to yield derivatives **8j** and **8k**, respectively, was detrimental for antiproliferatve activity against U-937 cells as compared to that of **2a**, in particular for the *p*-methoxybenzylamino derivative **8j**, while derivative **2a** and **8k** were equipotent against SK-MEL-1 cells. Moreover, the *p*-methoxyphenylethylamino derivative **8k** was more potent as an antiproliferative agent than the corresponding previously reported *p*-methoxyphenyl counterpart **2a** [20].

Comparing the activities of unsubstituted phenyl derivatives **8a**, **8b** and **8c**, increasing the length of the linker between the aryl moiety and the nitrogen at the 2-position of the thiazole ring from one (**8a**) to two (**8b**) to three (**8c**) methylene units, similar activity was observed for **8a** and **8b** and a 2-fold increase in potency occurred with **8c** against U-937 cells. While **8a** and **8c** were equipotent, a slight reduction of activity was observed for the phenylethylamino derivative **8b** relative to **8a** and **8c** against SK-MEL-1 cancer cells. While the 2-*N*-methylbenzylamino derivative **3a** had IC_50_ values lower than 200 μM against a panel of four different cancer cell lines [21], the corresponding *N*-desmethyl analogue **8a** was ten-fold more potent than **3a**.

The substitution pattern on the phenyl of the benzylamino or phenylethylamino moiety at the 2-position of the thiazole ring plays an important role in the antiproliferative activity. In this respect, we found that only the insertion of the electron-withdrawing chlorine at the *para*-position of the phenyl ring, to furnish compound **8e**, enhanced activity significantly relative to the unsubstituted benzylamino analogue **8a**, while a significant loss in activity was observed by the insertion of a *p*-fluorine atom (compound **8d**). Furthermore, the *p*-chlorophenethylamino homologue **8f** of derivative **8e** had similar activity against U-937 cells, while this latter derivative was 1.5-fold more potent than **8f** against SK-MEL-1 cells. As for the benzylamino derivatives **8a** and **8e**, the *p*-chlorophenylethylamino analog **8f** was significantly more potent than the corresponding unsubstituted derivative **8b**.

The replacement of the chlorine by a bromine (compounds **8e** and **8g**, respectively) caused a reduction of activity, but this was less dramatic with respect to what was observed for the fluorine derivative **8d**. Comparison of the halogenated compounds (**8e** vs. **8d** and **8g**) indicated that the order of influence of halogen atoms on antiproliferative activity was Cl > Br >> F.

The replacement of the electron-withdrawing bromine with a weak electron-releasing methyl group in the phenyl of the 5-benzylamino moiety (compounds **8g** and **8h**, respectively) resulted in the retention of antiproliferative activity, indicating that the bromine atom and methyl group are bioequivalent at the *para*-position of the phenyl ring. Starting from this latter compound, moving the methyl group from the *para*- to the *meta*-position (compounds **8h** and **8i**, respectively) also resulted in no loss of activity.

The position of the methoxy substituent on the phenyl ring influenced antiproliferative activity, with the isomeric *m*- and *o*-methoxybenzylamino analogues **8l** and **8m** having similar antiproliferative activities, and both were 2-fold more potent than their *p*-methoxy counterpart **8j**. For this latter compound, placing an additional methoxy group at the *meta*-position, to yield **8n**, as well as the methylendioxy derivative **8o**, resulted in compounds equipotent with the *p*-methoxy derivative **8j**. It should be noted that, although this latter derivative showed significantly reduced activity as compared with the *p*-methoxyphenylamino analogue **2a**, the insertion of an additional methylene unit, to furnish the *p*-methoxyphenylethylamino derivative **8k**, significantly increased (3-4-fold) antiproliferative activity relative to that of **8j**.

In order to investigate the selective antiproliferative activity of the new synthesized compounds, we tested representative derivatives **8f** and **8k** against peripheral blood lymphocytes (PBL) isolated from healthy donors. These two molecules showed poor cytotoxic activity (IC_50_ > 100 μM) against normal cells, thus indicating potential high selectivity and a good safety profile in vitro.

### 3.2. Tubulin Polymerization and CDK Inhibitory Activity Assays

To investigate whether the antiproliferative activities of these new compounds were related to an interaction with tubulin, the most active compounds of the series (**8f** and **8k**) were selected to determine their ability to inhibit tubulin polymerization, because of the strong tubulin inhibitory effects previously observed with compounds with general structure **2**. For comparison, CA-4 was also examined in contemporaneous experiments as a positive reference control. In this assay, CA-4 strongly inhibited tubulin polymerization with IC_50_ value of 1.2 μM, while **8f** and **8k** did not show any noteworthy inhibition at high concentrations (up to 20 μM). The lack of activity of compounds **8f** and **8k** suggests that the nitrogen substitution at the 2-position of the 4-amino-5-(3′,4′,5′-trimethoxybenzoyl)thiazole scaffold is critically involved in tubulin binding. The introduction of a methylene or ethylene spacer between the nitrogen at the 2-position of the thiazole ring and the phenyl ring resulted in increased flexibility of this part of the molecule, and this could be a factor that dramatically decreased the biological activity of compounds **8a**–**o** relative to compounds with general structure **2**, characterized by the present of more rigid 2-anilino moiety.

Derivatives **8f** and **8k** were also evaluated at a single concentration of 10 μM in an in vitro assay against CDK4, CDK6 and CDK9 and minimal effects were observed (enzyme inhibition ranging from 2 to 8%). From these results, we conclude that compounds **8f** and **8k** are inactive both as tubulin polymerization and CDK inhibitors, suggesting that these molecules exert their antiproliferative effects by interacting with alternative intracellular targets.

### 3.3. Flow Cytometric Analysis in Human Myeloid Leukemia Cells

Induction of apoptosis in cancer cells is recognized as an efficient strategy for cancer chemotherapy [1]. For this reason, it was decided to determine whether these compounds display their cytotoxic action through alterations in cell cycle progression and induction of cell apotosis. To this end, the effects at different time periods (6, 12 and 24 h) with different concentrations (10 and 30 μM) of **8f** and **8k** on cell cycle progression on U-937 leukemia cells were investigated by flow cytometry analyses. The percentages of cells in different phases of cell cycle are shown in Table 2.

As shown, both compounds caused an increase in the percentage of cells in both the G_1_ and S-phases and a small increase in the G_2_-M-phase. These effects were most marked with 30 µM compound at 24 h. The small effect on the proportion of cells in the G_2_-M phase confirmed the lack of microtubule activity observed in the tubulin polymerization assay. The detection of a sub-G_0_-G_1_ peak (indication of DNA degradation) by propidium iodide staining upon incubation with **8f** and **8k**, suggested that these compounds exert their growth inhibiting effect by induction of apoptosis. The proportion of apoptotic cells increased with incubation time and compound concentration.

**Figure 3:**
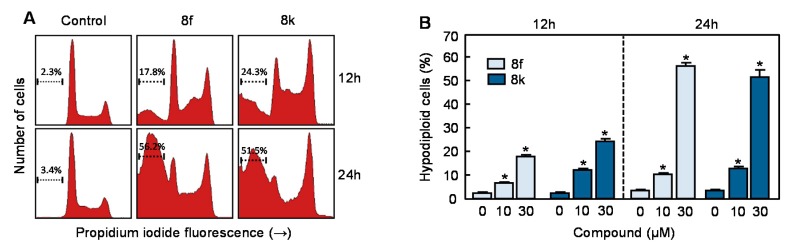
(**A**) U-937 cells were incubated with 30 μM **8f** or **8k** for the indicated times and subjected to DNA flow cytometry using propidium iodide labeling. Representative histograms and the percentage of hypodiploid cells (apoptotic cells) are shown. (**B**) U-937 cells were incubated with the indicated concentrations of **8f** or **8k** for the indicated times and the percentage of cells in the sub-G_1_ region was determined by flow cytometry. Error bars represent means ± SE of two independent experiments each performed in triplicate. * indicates *p* < 0.05 for comparison with untreated control.

Maximal levels of apoptotic cells, approximately 16.5-fold and 15-fold increases with respect to control cells were observed at 24 h with 30 μM **8f** or **8k** (Figure 3).

### 3.4. Molecular Modeling Studies

**Figure 4:**
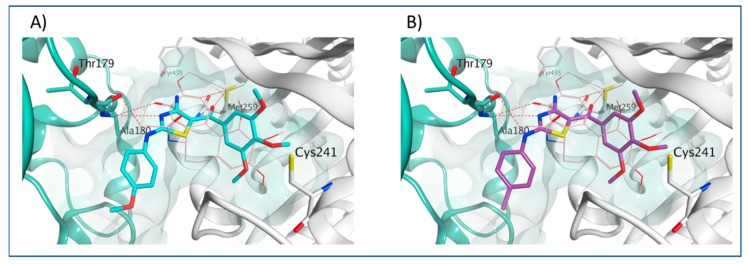
Proposed binding modes for compounds **2a** (**A**) and **2b** (**B**) in the colchicine site. The trimethoxyphenyl ring is oriented towards the α-tubulin subunit in proximity to βCys241, while the rest of the molecule forms three hydrogen bonds with αThr179, αAla180 and βMet259. Co-crystallized colchicine is shown in pink. The tubulin α-subunit is shown as a mint green ribbon, while the β-subunit is represented as a white ribbon.

A series of molecular docking simulations were performed on selected compounds (**2a**, **2b**, **8h**, **8f** and **8k**) in order to investigate their putative interaction with the colchicine binding site of tubulin. In the tubulin assembly assay, compound **2b** was found to be the most active (IC_50_, 0.72 μM) in the series of derivatives with general structure **2**, and it was twice as potent as CA-4 (IC_50_, 1.4 μM). Previously reported compounds **2a** and **2b** place their trimethoxyphenyl ring in the β-tubulin subunit close to βCys241, partially overlapping the co-crystallized colchicine. Hydrogen bond formation between the nitrogen at the 2-position of the thiazole ring and αThr179, the thiazole core and backbone of αAla180 and the carbonyl group and βMet259, with these two last residues also involved in the tubulin-colchicine interaction, contribute to stabilize the binding of the two molecules (Figure 4).

**Figure 5:**
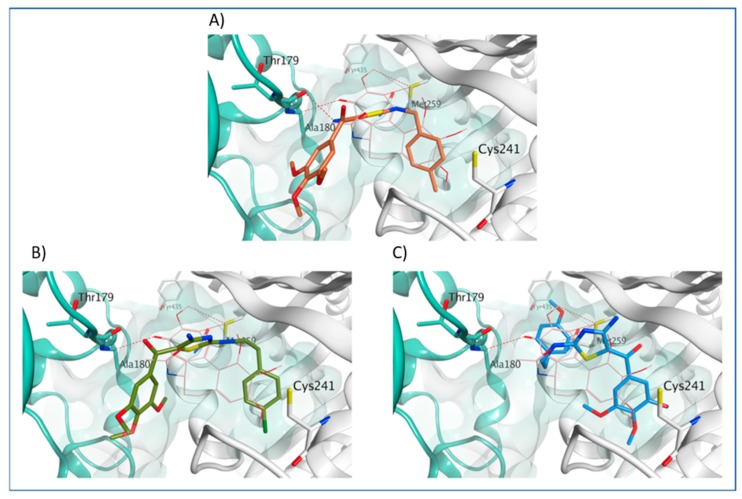
Proposed binding modes for compounds **8h** (**A**), **8f** (**B**) and **8k** (**C**) in the colchicine site. The increased flexibility introduced by the methylene or ethylene spacer causes an inconsistent binding. Co-crystallized colchicine is shown in pink. The tubulin α-subunit is shown as a mint green ribbon, while the β-subunit is represented as a white ribbon.

The increased flexibility introduced by the methylene (**8h**) or ethylene (**8f**, **8k**) spacer between the nitrogen at the 2-position of the thiazole ring and the phenyl ring causes an inconsistent binding of the compounds, which either occupy the active site in a different orientation, placing the trimethoxyphenyl ring away from βCys241 (Figure 5, **8h** and **8f** for Panels C and D, respectively) or adopt a non-optimal occupation of the binding area (Figure 5, Panel E for **8k**). In both cases, the inability to correctly occupy the colchicine binding site could lead to a lack of inhibition of tubulin polymerization.

## 4. Experimental

### 4.1. Chemistry

#### 4.1.1. Materials and Methods

^1^H-NMR spectra were recorded on either an AC 200 (Bruker, Bremen, Germany) or a 400 Mercury Plus (Varian, Palo Alto, CA, USA) spectrometer, while ^13^C-NMR spectra were recorded on the Varian 400 Mercury Plus spectrometer. Chemical shifts (δ) are given in ppm upfield, and the spectra were recorded in appropriate deuterated solvents, as indicated. All products reported showed ^1^H- and ^13^C-NMR spectra in agreement with the assigned structures. Positive-ion electrospray ionization (ESI) mass spectra were recorded on a double-focusing Finnigan MAT 95 instrument (Finnigan, Waltham, MA, USA) with BE geometry. Melting points (mp) were determined on a Buchi-Tottoli apparatus (Buchi, Milan, Italy) and are uncorrected. The purity of tested compounds was determined by combustion elemental analyses conducted by the Microanalytical Laboratory of the Chemistry Department of the University of Ferrara with a MT-5 CHN recorder elemental analyzer (Yanagimoto, Kyoto, Japan). All tested compounds yielded data consistent with a purity of at least 95% as compared with the theoretical values. Reaction courses and product mixtures were routinely monitored by thin layer chromatography on silica gel (precoated F254 plates, Merck, Darmstadt, Germany), and compounds were visualized with aqueous KMnO_4_. Flash chromatography was performed using 230–400 mesh silica gel and the indicated solvent system.

#### 4.1.2. General Procedure A for the Synthesis of Compounds **10a**–**o**

To a solution of dimethyl cyanodithioimidocarbonate **9** (292 mg, 2 mmol) in isopropanol (10 mL) was added the appropriate amine (2 mmol, 1 equiv.), and the mixture was stirred for 24 h at room temperature. After this time, the suspension was filtered, and the solid residue was washed with ethyl ether to furnish the final compound **10a–o** that was used for the next reaction without further purification.

##### (*Z*)-Methyl N-benzyl-*N*′-cyanocarbamimidothioate (**10a**)

Following general procedure A, compound **10a** was obtained as a white solid, yield > 95%, mp 156–158 °C. ^1^H-NMR (DMSO-***d_6_***) δ: 2.62 (s, 3H), 4.49 (s, 2H), 7.20–7.24 (m, 2H), 7.32–7.36 (m, 3H), 8.85 (bs, 1H). MS (ESI): [M + 1]^+^ = 206.2.

##### (*Z*)-Methyl N′-cyano-*N*-phenethylcarbamimidothioate (**10b**)

Following general procedure A, compound **10b** was obtained as a white solid, yield > 95%, mp 174–176 °C. ^1^H-NMR (CDCl_3_) δ: 2.46 (s, 3H), 2.91 (t, ***J*** = 7.2 Hz, 2H), 3.60 (bs, 2H), 7.20 (d, *J* = 6.8 Hz, 2H), 7.22–7.25 (m, 1H), 7.34 (t, *J* = 6.8 Hz. 2H). MS (ESI): [M + 1]^+^ = 220.6.

##### (*Z*)-Methyl N′-cyano-*N*-(3-phenylpropyl)carbamimidothioate (**10c**)

Following general procedure A, derivative **10c** was obtained as a white solid, yield > 95%, mp 180–182 °C. ^1^H-NMR (CDCl_3_) δ: 2.07–2.14 (m, 2H), 2.53 (bs, 3H), 2.81 (t, *J* = 9.6 Hz, 2H), 3.48 (bs, 2H), 7.30–7.44 (m, 5H). MS (ESI): [M + 1]^+^ = 234.5.

##### (*Z*)-methyl *N*-4-fluorobenzyl-*N*′-cyanocarbamimidothioate (**10d**)

Following general procedure A, compound **10d** was obtained as a yellow solid, yield 52%, mp 170–172 °C. ^1^H-NMR (CDCl_3_) δ: 2.53 (s, 3H), 4.52 (bs, 2H), 7.07 (t, *J* = 8.8 Hz, 2H), 7.30 (dd, *J* = 8.8 and 5.2 Hz, 2H). MS (ESI): [M + 1]^+^ = 224.4.

##### (*Z*)-methyl *N*-4-chlorobenzyl-*N′*-cyanocarbamimidothioate (**10e**)

Following general procedure A, compound **10e** was obtained as a yellow solid, yield 92%, mp 190–192 °C. ^1^H-NMR (CDCl_3_) δ: 2.38 (s, 3H), 4.42 (bs, 2H), 7.02 (d, *J* = 8.0 Hz, 2H), 7.20 (d, *J* = 8.0 Hz, 2H). MS (ESI): [M + 1]^+^ = 240.2.

##### (*Z*)-Methyl *N*-4-chlorophenethyl-*N′*-cyanocarbamimidothioate (**10f**)

Following general procedure A, compound **10f** was obtained as a yellow solid, yield 86%, mp 148–150 °C. ^1^H-NMR (CDCl_3_) δ: 2.48 (s, 3H), 2.92 (t, *J* = 6.8 Hz, 2H), 3.62 (bs, 2H), 7.14 (d, *J* = 8.4 Hz, 2H), 7.31 (d, *J* = 8.4 Hz, 2H). MS (ESI): [M + 1]^+^ = 254.3.

##### (*Z*)-methyl *N*-4-bromobenzyl-*N′*-cyanocarbamimidothioate (**10g**)

Following general procedure A, compound **10g** was obtained as a yellow solid, yield 91%, mp 199–201 °C. ^1^H-NMR (DMSO-*d_6_*) δ: 2.62 (s, 3H), 4.45 (bs, 2H), 7.24 (bs, 2H), 7.24 (d, *J* = 8.8 Hz, 2H), 7.53 (d, *J* = 8.8 Hz, 2H), 8.76 (bs, 1H). MS (ESI): [M]^+^ = 234.1 and 236.1.

##### (*Z*)-Methyl *N′*-cyano-*N*-(4-methylbenzyl)carbamimidothioate (**10h**)

Following general procedure A, compound **10h** was obtained as a white solid, yield 80%, mp 192–194 °C. ^1^H-NMR (DMSO-*d_6_*) δ: 2.26 (s, 3H), 2.59 (s, 3H), 4.41 (bs, 2H), 7.12 (d, *J* = 8.2 Hz, 2H), 7.15 (d, *J* = 8.2 Hz, 2H), 8.83 (bs, 1H). MS (ESI): [M + 1]^+^ = 220.3.

##### (*Z*)-Methyl *N′*-cyano-*N*-(3-methylbenzyl)carbamimidothioate (**10i**)

Following general procedure A, compound **10i** was obtained as a white solid, yield 61%, mp 178–180 °C. ^1^H-NMR (DMSO-*d_6_*) δ: 2.30 (s, 3H), 2.64 (s, 3H), 4.45 (bs, 2H), 7.04–7.10 (m, 3H), 7.21 (t, *J* = 6.8 Hz, 1H), 8.83 (bs, 1H). MS (ESI): [M + 1]^+^ = 220.2.

##### (*Z*)-Methyl *N′*-cyano-*N*-(4-methoxybenzyl)carbamimidothioate (**10j**)

Following general procedure A, compound **10j** was obtained as a white solid, yield > 95%, mp 161–163 °C. ^1^H-NMR (DMSO-*d_6_*) δ: 2.59 (s, 3H), 3.73 (s, 3H), 4.40 (bs, 2H), 6.90 (d, *J* = 8.8 Hz, 2H), 7.22 (d, *J* = 8.8 Hz, 2H), 8.87 (bs, 1H). MS (ESI): [M + 1]^+^ = 236.3.

##### (*Z*)-Methyl *N*-4-methoxyphenethyl-*N′*-cyanocarbamimidothioate (**10k**)

Following general procedure A, compound **10k** was obtained as a yellow solid, yield > 95%, mp 168–170 °C. ^1^H-NMR (CDCl_3_) δ: 2.45 (s, 3H), 2.87 (t, *J* = 6.8 Hz, 2H), 3.58 (bs, 2H), 3.82 (s, 3H), 6.88 (d, *J* = 8.8 Hz, 2H), 7.11 (d, *J* = 8.8 Hz, 2H). MS (ESI): [M + 1]^+^ = 260.3.

##### (*Z*)-Methyl *N′*-cyano-*N*-(3-methoxybenzyl)carbamimidothioate (**10l**)

Following general procedure A, compound **10l** was obtained as a white solid, yield: 89%, mp 146–148 °C. ^1^H-NMR (CDCl_3_) δ: 2.46 (s, 3H), 3.83 (s, 3H), 4.44 (bs, 2H), 6.84 (s, 1H), 6.88 (dd, *J* = 7.6 and 2.4 Hz, 2H), 7.29 (d, *J* = 7.6 Hz, 1H). MS (ESI): [M + 1]^+^ = 236.5.

##### (*Z*)-Methyl *N′*-cyano-*N*-(2-methoxybenzyl)carbamimidothioate (**10m**)

Following general procedure A, compound **10m** was obtained as a white solid, yield > 95%, mp 160–162 °C. ^1^H-NMR (CDCl_3_) δ: 2.46 (s, 3H), 3.82 (s, 3H), 4.44 (bs, 2H), 6.92–6.97 (m, 3H), 7.35 (t, *J* = 8.2 Hz, 1H). MS (ESI): [M + 1]^+^ = 236.2.

##### (*Z*)-methyl *N′*-cyano-*N*-(3,4-dimethoxybenzyl)carbamimidothioate (**10n**)

Following general procedure A, compound **10n** was obtained as a white solid, yield 89%, mp 196–198 °C. ^1^H-NMR (CDCl_3_) δ: 2.53 (s, 3H), 3.90 (s, 3H), 3.91 (s, 3H), 4.47 (bs, 2H), 6.80 (s, 1H), 6.87 (d, *J* = 1.2 Hz, 2H). MS (ESI): [M + 1]^+^ = 266.4.

##### (*Z*)-Methyl *N*-(benzo[d][1,3]dioxol-5-ylmethyl)-*N′*-cyanocarbamimidothioate (**10o**)

Following general procedure A, derivative **10o** was obtained as a white solid, yield 81%, mp 188–190 °C. ^1^H-NMR (CDCl_3_) δ: 2.42 (s, 3H), 4.42 (bs, 2H), 5.99 (s, 2H), 6.76–6.82 (m, 3H). MS (ESI): [M + 1]^+^ = 250.2

#### 4.1.3. General Procedure B for the Synthesis of Compounds **8a**–**o**

To a stirred solution of the appropriate (*Z*)-methyl *N*-arylalkyl-*N’*-cyanocarbamimidothioate **10a**–**o** (1 mmol) in DMF (5 mL), heated at 80 °C, Na_2_S 9 H_2_O (240 mg, 1 mmol) was added, and the mixture was heated for 90 min at 80 °C. Then, 2-bromo-1-(3′,4′,5′-trimethoxyphenyl)ethanone (580 mg, 2 mmol) dissolved in DMF (3 mL) was slowly added dropwise at 50 °C. The mixture was heated at 50 °C for 2 h, and potassium carbonate (276 mg, 2 mmol) was added. The reaction was stirred at 50 °C for an additional 1 h. The mixture was poured into water (20 mL), and the resulting suspension was extracted with dichloromethane (3 × 15 mL). The combined organic phases were washed with water (2 × 15 mL) and brine (20 mL), dried over Na_2_SO_4_ and concentrated under reduced pressure. The resulting residue was purified by column chromatography on silica gel.

#### (4-Amino-2-(benzylamino)thiazol-5-yl)(3,4,5-trimethoxyphenyl)methanone (**8a**)

Following general procedure B, the crude residue purified by flash chromatography, using EtOAc: petroleum ether 1:1 (*v*:*v*) for elution, furnished **8a** as a yellow solid. Yield 32%, mp 147–149 °C. ^1^H-NMR (CDCl_3_) δ: 3.87 (s, 6H), 3.90 (s, 3H), 4.49 (bs, 2H), 6.98 (s, 2H), 7.33–7.36 (m, 4H). ^13^C-NMR (CDCl_3_) δ: 48.89, 55.63 (2C), 60.34, 92.62, 104.07 (2C), 106.61, 127.15 (2C), 127.79, 128.44 (2C), 135.05, 139.55, 152.46 (2C), 162.84, 171.24, 183.14. MS (ESI): [M + 1]^+^ = 400.3. Anal. calcd for C_20_H_21_N_3_O_4_S. C, 60.13; H, 5.30; N, 10.52; found: C, 59.87; H, 5.17; N, 10.26.

#### (4-Amino-2-(phenethylamino)thiazol-5-yl)(3,4,5-trimethoxyphenyl)methanone (**8b**)

Following general procedure B, the crude residue purified by flash chromatography, using EtOAc: petroleum ether 6:4 (*v*:*v*) for elution, furnished **8b** as a yellow solid. Yield 39%. Mp 135–138 °C. ^1^H-NMR (CDCl_3_) δ: 2.99 (t, 2H), 3.58 (t, 2H), 3.85 (s, 9H), 6.99 (s, 2H), 7.22 (d, *J* = 8.0 Hz, 2H), 7.35–7.40 (m, 3H). ^13^C-NMR (CDCl_3_) δ: 34.98, 46.96, 56.26 (2C), 60.92, 104.64 (2C), 127.05 (2C), 128.72 (2C), 128.91 (2C), 136.52, 137.42 (2C), 140.11. 153.05 (2C), 171.50, 183.73. MS (ESI): [M + 1]^+^ = 414.49. Anal. calcd for C_21_H_23_N_3_O_4_S. C, 61.00; H, 5.61; N, 10.16; found: C, 59.88; H, 5.37; N, 9.85.

#### (4-Amino-2-((3-phenylpropyl)amino)thiazol-5-yl)(3,4,5-trimethoxyphenyl)methanone (**8c**)

Following general procedure B, the crude residue purified by flash chromatography, using EtOAc: petroleum ether 6:4 (*v*:*v*) for elution, furnished **8c** as a yellow oil. Yield 37%. ^1^H-NMR (CDCl_3_) δ: 2.00–2.07 (m, 2H), 2.75 (t, *J* = 8.0 Hz, 2H), 3.30 (t, *J* = 8.0 Hz, 2H), 3.91 (s, 9H), 6.99 (s, 2H), 7.19 (d, *J* = 8.0 Hz, 2H), 7.28–7.32 (m, 3H). ^13^C-NMR (CDCl_3_) δ: 30.20, 32.91, 45.58, 56.32 (2C), 61.03, 104.71 (2C), 107.28, 126.45, 128.45 (2C), 128.59 (2C), 128.71 (2C), 136.37, 140.40, 153.04, 153.19, 171.19, 183.96. MS (ESI): [M + 1]^+^ = 428.15. Anal. calcd for C_22_H_25_N_3_O_4_S. C, 61.81; H, 5.89; N, 9.83; found: C, 61.54; H, 5.32; N, 9.68.

#### (4-Amino-2-((4-fluorobenzyl)amino)thiazol-5-yl)(3,4,5-trimethoxyphenyl)methanone (**8d**)

Following general procedure B, the crude residue purified by flash chromatography, using EtOAc: petroleum ether 6:4 (*v*:*v*) for elution, furnished **8d** as an orange solid. Yield 42%, mp 132–134 °C. ^1^H-NMR (CDCl_3_) δ: 3.89 (s, 6H), 3.90 (s, 3H), 4.43 (bs, 2H), 6.40 (bs, 2H), 7.00 (s, 2H), 7.17 (t, *J* = 8.0 Hz, 2H), 7.35–7.40 (m, 2H). ^13^C-NMR (CDCl_3_) δ: 49.23, 56.23 (2C), 60.90, 105.48 (2C), 112.09, 128.33 (2C), 128.78, 129.52 (2C), 135.56, 137.24, 140.30, 153.02 (2C), 161.32, 164.34, 184.57. MS (ESI): [M + 1]^+^ = 410.8. Anal. calcd for C_20_H_20_FN_3_O_4_S. C, 57.54; H, 4.83; N, 10.07; found: C, 57.37; H, 4.67; N, 9.89.

#### (4-Amino-2-((4-chlorobenzyl)amino)thiazol-5-yl)(3,4,5-trimethoxyphenyl)methanone (**8e**)

Following general procedure B, the crude residue purified by flash chromatography, using EtOAc: petroleum ether 1:1 (*v*:*v*) for elution, furnished **8e** as a yellow solid. Yield 39%, mp 156–159 °C. ^1^H-NMR (CDCl_3_) δ: 3.86 (s, 6H), 3.88 (s, 3H), 4.47 (bs, 2H), 6.93 (s, 2H), 7.27 (d, *J* = 8.0 Hz, 2H), 7.35 (d, *J* = 8.0 Hz, 2H). ^13^C-NMR (CDCl_3_) δ: 49.06, 56.30 (2C), 61.03, 104.67 (2C), 107.34, 129.12, 129.24 (2C), 129.29 (2C), 133.34, 134.54, 135.78, 140.60, 153.26 (2C), 170.14, 184.23. MS (ESI): [M + 1]^+^ = 434.62. Anal. calcd for C_20_H_20_ClN_3_O_4_S. C, 55.36; H, 4.65; N, 9.68; found: C, 55.02; H, 4.39; N, 9.47.

#### (4-Amino-2-((4-chlorophenethyl)amino)thiazol-5-yl)(3,4,5-trimethoxyphenyl)methanone (**8f**)

Following general procedure B, the crude residue purified by flash chromatography, using EtOAc: petroleum ether 6:4 (*v*:*v*) for elution, furnished **8f** as an orange solid. Yield 31%. ^1^H-NMR (CDCl_3_) δ: 2.94 (t, *J* = 8.0 Hz, 2H), 3.55 (t, *J* = 8.0 Hz, 2H), 3.91 (s, 9H), 5.80 (bs, 1H), 7.02 (s, 2H), 7.14 (d, *J* = 8.0 Hz 2H), 7.30 (d, *J* = 8.0 Hz 2H). ^13^C-NMR (CDCl_3_) δ: 34.50, 46.22, 56.22 (2C), 60.91, 104.66 (2C), 129.01 (2C), 130.06 (2C), 132.91, 136.06 (2C), 136.87, 140.02, 152.98 (2C), 165.06, 172.08, 183.67. MS (ESI): [M + 1]^+^ = 448.39. Anal. calcd for C_21_H_22_ClN_3_O_4_S. C, 56.31; H, 4.95; N, 9.38; found: C, 56.01; H, 4.73; N, 9.07.

#### (4-Amino-2-((4-bromobenzyl)amino)thiazol-5-yl)(3,4,5-trimethoxyphenyl)methanone (**8g**)

Following general procedure B, the crude residue purified by flash chromatography, using EtOAc: petroleum ether 1:1 (*v*:*v*) for elution, furnished **8g** as a yellow solid. Yield 31%, mp 154–157 °C. ^1^H-NMR (CDCl_3_) δ: 3.86 (s, 6H), 3.88 (s, 3H), 3.92 (bs, 1H), 4.44 (bs, 2H), 6.95 (s, 2H), 7.20 (d, *J* = 8.4 Hz, 2H), 7.47 (d, *J* = 8.4 Hz, 2H). ^13^C-NMR (CDCl_3_) δ: 48.63, 56.17 (2C), 60.90, 104.62 (2C), 110.00, 122.24, 129.31 (2C), 132.07 (2C), 134.74, 136.39, 140.17, 153.03 (2C), 163.20, 171.52, 183.79. MS (ESI): [M + 1]^+^ = 434.09. Anal. calcd for C_20_H_20_BrN_3_O_4_S. C, 20.22; H, 4.21; N, 8.78; found: C, 20.01; H3.99; N, 8.57.

#### (4-Amino-2-((4-methylbenzyl)amino)thiazol-5-yl)(3,4,5-trimethoxyphenyl)methanone (**8h**)

Following general procedure B, the crude residue purified by flash chromatography, using EtOAc: petroleum ether 1:1 (*v*:*v*) for elution, furnished **8h** as a yellow solid. Yield 36%, mp 145–147 °C. ^1^H-NMR (CDCl_3_) δ: 2.36 (s, 3H), 3.89 (s, 6H), 3.90 (s, 3H), 3.94 (bs 1H) 4.44 (bs, 2H), 6.36 (bs, 2H), 7.01 (s, 2H), 7.19 (d, *J* = 8.0 Hz, 2H), 7.22 (d, *J* = 8.0 Hz, 2H). ^13^C-NMR (CDCl_3_) δ: 21.22, 49.23, 56.26 (2C), 60.99, 104.75 (2C), 110.09, 127.79 (2C), 128.83, 129.74 (2C), 132.96, 136.90, 138.28, 153.05 (2C), 162.32, 173.34, 183.69. MS (ESI): [M + 1]^+^ = 414.3. Anal. calcd for C_21_H_23_N_3_O_4_S. C, 61.00; H, 5.61; N, 10.16; found: C, 60.65; H, 5.28; N, 10.01.

#### (4-Amino-2-((3-methylbenzyl)amino)thiazol-5-yl)(3,4,5-trimethoxyphenyl)methanone (**8i**)

Following general procedure B, the crude residue purified by flash chromatography, using EtOAc:petroleum ether 4:6 (*v*:*v*) for elution, furnished **8i** as a yellow solid. Yield 48%, mp 150–152 °C. ^1^H-NMR (CDCl_3_) δ: 2.37 (s, 3H), 3.89 (s, 6H), 3.90 (s, 3H), 4.45 (bs, 2H), 7.01 (s, 2H), 7.13–7.15 (m, 2H), 7.20–7.23 (m, 2H). ^13^C-NMR (CDCl_3_) δ: 21.47, 45.35, 56.27 (2C), 60.99, 104.76 (2C), 124.80, 128.44, 128.97, 129.13 (2C), 135.99, 136.91, 138.92, 140.08, 153.05 (2C), 165.04, 172.34, 183.73. MS (ESI): [M + 1]^+^ = 414.2. Anal. calcd for C_21_H_23_N_3_O_4_S. C, 61.00; H, 5.61; N, 10.16; found: C, 60.72; H, 5.33; N, 10.04.

#### (4-Amino-2-((4-methoxybenzyl)amino)thiazol-5-yl)(3,4,5-trimethoxyphenyl)methanone (**8j**)

Following general procedure B, the crude residue purified by flash chromatography, using EtOAc: petroleum ether 1:1 (*v*:*v*) for elution, furnished **8j** as a yellow solid. Yield 40%, mp 131–135 °C. ^1^H-NMR (CDCl_3_) δ: 3.81 (s, 3H), 3.89 (s, 6H), 3.90 (s, 3H), 3.93 (bs, 1H) 4.42 (bs, 2H), 6.89 (d, *J* = 8.4 Hz, 2H), 6.98 (s, 2H), 7.27 (d, *J* = 8.8 Hz, 2H). ^13^C-NMR (CDCl_3_) δ: 49.16, 55.41, 56.29 (2C), 61.01, 104.71 (2C), 107.29, 114.43 (2C), 127.30, 129.32 (2C), 132.34, 136.41, 140.12, 153.14 (2C), 159.77, 171.24, 183.87. MS (ESI): [M + 1]^+^ = 430.27. Anal. calcd for C_21_H_23_N_3_O_5_S. C, 58.73; H, 5.40; N, 9.78; found: C, 58.55; H, 5.13; N, 9.47.

#### (4-Amino-2-((4-methoxyphenethyl)amino)thiazol-5-yl)(3,4,5-trimethoxyphenyl)methanone (**8k**)

Following general procedure B, the crude residue purified by flash chromatography, using EtOAc: petroleum ether 6:4 (*v*:*v*) for elution, furnished **8k** as a yellow solid. Yield 29%. ^1^H-NMR (CDCl_3_) δ: 2.92 (t, *J* = 6.8 Hz, 2H), 3.52 (t, J = 6.8 Hz, 2H), 3.81 (s, 3H), 3.91 (s, 9H), 5.80 (bs, 1H), 6.89 (d, *J* = 8.0 Hz, 2H), 7.02 (s, 2H), 7.14 (d, *J* = 8.0 Hz 2H). ^13^C-NMR (CDCl_3_) δ: 34.27, 46.74, 55.39, 56.30, 56.42, 60.99, 104.74 (2C), 105.54, 114.30, 114.41 (2C), 129.50, 129.78 (2C), 136.99, 140.07, 153.06, 158.74, 172.30, 183.68. MS (ESI): [M + 1]^+^ = 444.21. Anal. calcd for C_21_H_25_N_3_O_5_S. C, 59.58; H, 5.68; N, 9.47; found: C, 59.21; H, 5.33; N, 9.19.

#### (4-Amino-2-((3-methoxybenzyl)amino)thiazol-5-yl)(3,4,5-trimethoxyphenyl)methanone (**8l**)

Following general procedure B, the crude residue purified by flash chromatography, using EtOAc: petroleum ether 6:4 (*v*:*v*) for elution, furnished **8l** as an orange solid. Yield 40%, mp 125–128 °C. ^1^H-NMR (CDCl_3_) δ: 3.81 (s, 3H), 3.89 (s, 9H), 3.91 (bs, 1H), 4.45 (bs, 2H), 6.85–6.90 (m, 2H), 6.99 (s, 2H), 7.29 (d, *J* = 8.0 Hz, 2H). ^13^C-NMR (CDCl_3_) δ: 49.39, 55.36, 56.26 (2C), 60.97, 93.74, 104.73 (2C), 107.22, 113.53 (2C), 119.88, 130.14, 136.76, 137.49, 140.11, 153.06, 160.14, 164.44, 172.32, 183.68. MS (ESI): [M + 1]^+^ = 430.20. Anal. calcd for C_21_H_23_N_3_O_5_S. C, 58.73; H, 5.40; N, 9.78; found: C, 58.55; H, 5.17; N, 9.61.

#### (4-Amino-2-((2-methoxybenzyl)amino)thiazol-5-yl)(3,4,5-trimethoxyphenyl)methanone (**8m**)

Following general procedure B, the crude residue purified by flash chromatography, using EtOAc: petroleum ether 6:4 (*v*:*v*) for elution, furnished **8m** as an orange solid. Yield 42%. ^1^H-NMR (CDCl_3_) δ: 3.88 (s, 3H), 3.93 (s, 9H), 4.47 (bs, 2H), 6.92–6.95 (m, 2H), 6.98 (s, 2H), 7.35 (d, *J* = 8.0 Hz, 2H). ^13^C-NMR (CDCl_3_) δ: 46.12, 55.52, 56.31 (2C), 61.01, 104.70 (2C), 107.31, 110.68, 120.63 (2C), 123.18, 129.74 (2C), 130.09, 136.27, 140.36, 153.17, 157.57, 170.82, 183.95. MS (ESI): [M + 1]^+^ = 430.22. Anal. calcd for C_21_H_23_N_3_O_5_S. C, 58.73; H, 5.40; N, 9.78; found: C, 58.49; H, 5.22; N, 9.55.

#### (4-Amino-2-((3,4-dimethoxybenzyl)amino)thiazol-5-yl)(3,4,5-trimethoxyphenyl)methanone (**8n**)

Following general procedure B, the crude residue purified by flash chromatography, using EtOAc: petroleum ether 6:4 (*v*:*v*) for elution, furnished **8n** as a brown solid. Yield 39%, mp 107–111 °C. ^1^H-NMR (CDCl_3_) δ: 3.82 (s, 3H), 3.86 (s, 3H), 3.89 (s, 6H), 3.91 (s, 3H), 4.43 (bs, 2H), 6.81–6.93 (m, 3H), 6.97 (s, 2H). ^13^C-NMR (CDCl_3_) δ: 15.34, 49.74, 56.02, 56.12, 56.31, 61.01, 65.92, 104.69 (2C), 107.32, 111.01 (2C), 111.22 (2C), 120.56 (2C), 140.57, 149.35, 149.57, 153.04, 153.23, 184.14. MS (ESI): [M + 1]^+^ = 460.52. Anal. calcd for C_22_H_25_N_3_O_6_S. C, 57.50; H, 5.48; N, 9.14; found: C, 57.32; H, 5.19; N, 8.92.

#### (4-Amino-2-((benzo[d][1,3]dioxol-5-ylmethyl)amino)thiazol-5-yl)(3,4,5-trimethoxyphenyl) methanone (**8o**)

Following general procedure B, the crude residue purified by flash chromatography, using EtOAc: petroleum ether 6:4 (*v*:*v*) for elution, furnished **8o** as an orange solid. Yield 32%, mp 156–158 °C. ^1^H-NMR (CDCl_3_) δ: 3.88 (s, 6H), 3.90 (s, 3H), 4.40 (bs, 2H), 6.00 (s, 2H), 6.78–6.80 (m, 3H), 6.99 (s, 2H), 7.20–7.23 (m, 2H). ^13^C-NMR (CDCl_3_) δ: 49.21, 56.32 (2C), 60.89, 104.75 (2C), 100.3, 107.54, 110.09, 120.33, 133.75, 136.13, 137.48, 140.33, 141.44, 145.72, 146.80, 153.05 (2C), 163.32, 184.75. MS (ESI): [M + 1]^+^ = 444.2. Anal. calcd for C_21_H_21_N_3_O_6_S. C, 56.87; H, 4.77; N, 9.48; found: C, 56.69; H, 4.58; N, 9.28.

### 4.2. Biological Evaluation

#### 4.2.1. Cell Culture and Cell Viability Assays

Human U-937 and SK-MEL-1 cell lines were from the German Collection of Microorganisms and Cell Cultures (Braunschweig, Germany; DSMZ No: ACC 5 and ACC 303, respectively). The U-937 is a myeloid leukemia cell line isolated from a histiocytic lymphoma. SK-MEL-1 is a melanoma cell line. Cells were cultured in suspension in RPMI 1640 medium supplemented with 10% (*v*/*v*) fetal bovine serum and antibiotics (100 μg mL^−1^ streptomycin and 100 U mL^−1^ penicillin), at 37 °C in a humidified atmosphere containing 5% CO_2_. The trypan blue exclusion method was used for counting the cells by a hemocytometer, and cell viability was always greater than 95% in all experiments. Compounds were dissolved in DMSO, and further dilutions were made in culture medium just before use. The final concentration of DMSO did not exceed 0.3% (*v*/*v*) in any experiment. The effects on cell viability of synthetic compounds were determined using a colorimetric assay with the reduction of 3-(4,5-dimethyl-2-thiazolyl-)-2,5-diphenyl-2*H*-tetrazolium bromide (MTT) as previously described [28]. Briefly, 5 × 10^3^ cells were seeded in 96-well microculture plates with increasing concentrations of compounds for 72 h. Then, MTT (0.5 mg mL^−1^) was added and incubated for 4 h, and the reaction was stopped with 20% sodium dodecyl sulfate. A_570_ was used as a measure of cell viability. Concentrations inducing a 50% inhibition of cell growth were determined graphically by nonlinear regression using the curve-fitting algorithm of GraphPad Prism 4.0 (GraphPad Inc La Jolla, CA, USA). In all cytotoxicity assays, etoposide, doxorubicin and CA-4 were included as positive controls.

PBLs from healthy donors were obtained by separation on Lymphoprep (Fresenius KABI Norge AS, Halden, Norway) gradient. After extensive washing, cells were resuspended (1.0 × 10^6^ cells/mL) in RPMI-1640 with 10% fetal bovine serum and incubated overnight in a 96-well tissue culture microtiter plate. Varying compound concentrations were added to the cells, and the MTT assay was used to assess viability after a 48 h treatment.

#### 4.2.2. Analysis of Cell Cycle by Flow Cytometry

Flow cytometric analysis of propidium iodide-stained cells was performed as previously described [29]. Briefly, U-937 cells were treated with the indicated compounds at the specified concentrations in RPMI 1640 medium. After treatments, cells were centrifuged for 10 min at 500× *g,* washed with cold phosphate-buffered saline (PBS), fixed with ice-cold 75% ethanol and stored at −20 °C for at least 1 h. Samples were then centrifuged at 500 × *g* for 10 min at 4 °C, washed with PBS, resuspended in 200 μL of PBS containing 100 μg mL^−1^ RNase A and 50 μg mL^−1^ propidium iodide and incubated for 1 h in the dark. The DNA content was analyzed by flow cytometry with a Becton-Dickinson FACSVerse^TM^ cytometer (BD Biosciences, San Jose, CA, USA). The percentage of cells in each phase of the cell cycle was determined for a minimum of 10^4^ cells, and histograms were analyzed with Flowing 2.5.0 software.

#### 4.2.3. Effects on Tubulin Polymerization

Bovine brain tubulin was purified as described previously [30]. To evaluate the effect of the compounds on tubulin assembly in vitro [31], varying concentrations were preincubated with 10 μM tubulin in glutamate buffer at 30 °C and then cooled to 0 °C. After addition of GTP, the mixtures were transferred to 0 °C cuvettes in a recording spectrophotometer and warmed to 30 °C, and the assembly of tubulin was observed turbidimetrically. The IC_50_ was defined as the compound concentration that inhibited the extent of assembly by 50% after a 20 min incubation.

#### 4.2.4. In Vitro CDK Inhibition Assay

The CDK4, 6 and 9 inhibitory activies were determined using the CDK4/Cyclin D1 Assay kit (ProQinase, Freiburg im Breisgau, Germany), CDK6/Cyclin D3 Kinase Enzyme System (Promega, Milan, Italy) and CDK9/CyclinK Kinase Enzyme System (Promega) by ADP-Glo CDK assays following the manufacturer’s instructions. The assay was performed with the test compounds at 10 μM.

#### 4.2.5. Molecular Modeling Methods

All molecular docking studies were performed on a Viglen Genie Intel^®^CoreTM i7-3770 vPro CPU@ 3.40 GHz × 8 running Ubuntu 18.04. Molecular Operating Environment (MOE) 2019.10 [32] and Maestro (Schrödinger Release 2019-3) [33] were used as molecular modeling software. The tubulin structure was downloaded from the PDB data bank (http://www.rcsb.org/; PDB code 4O2B). The protein was pre-processed using the Schrödinger Protein Preparation Wizard by assigning bond orders, adding hydrogens and performing a restrained energy minimization of the added hydrogens using the OPLS_2005 force field. Ligand structures were built with MOE and then prepared using the Maestro LigPrep tool by energy minimizing the structures (OPLS_2005 force field), generating possible ionization states at pH 7 ± 2 and low-energy ring conformers. After isolating a tubulin dimer structure, a 12 Å docking grid (inner-box 10 Å and outer-box 22 Å) was prepared using as centroid the co-crystallized colchicine. Molecular docking studies were performed using Glide SP precision, keeping the default parameters and setting 10 as the number of output poses per input ligand to include in the solution. The output database was saved as a mol2 file. The docking results were visually inspected for the ability of the analyzed compounds to bind in the active site.

#### 4.2.6. Statistical Methods

Statistical differences between means of control and treated samples were tested using Student’s *t*-test. A significance level of *p* < 0.05 was used.

## 5. Conclusions

In the work described herein, we have reported a new series of compounds, characterized by a common 4-amino-5-(3′,4′,5′-trimethoxybenzoyl)thiazole moiety and modified at the 2-position of thiazole ring, designed to determine the potential of incorporating in a single molecule both CDK inhibition and antitubulin activity by a pharmacophore fusion approach. Three of the synthesized compounds, corresponding to *para*-chlorobenzylamino, *para*-chloro and *para*-methoxyphenethyl-amino analogues **8e**, **8f** and **8k**, respectively, were more active than the rest of the derivatives. The effects of the two latter compounds **8f** and **8k** on cell growth inhibition seemed to be due to the induction of apoptosis, showing significant apoptotic activity after a 24 h treatment at 30 μM, while **8f** and **8k** were inactive as dual inhibitors of tubulin polymerization and CDKs. Therefore, we assume that **8f** and **8k** exert their antiproliferative effect by a mechanism directed at one or more other cellular targets.

## Figures and Tables

**Table 1:** In vitro inhibitory effects of compounds **8a**–**o** and reference derivatives **1a**, **2a**, CA-4, doxorubicin and etoposide on proliferation of U-937 and SK-MEL-1 human tumor cell lines.

Compd	IC_50_ (μM) ^a^	
	U-937	SK-MEL-1
**8a**	21.1 ± 5.1	14.2 ± 9.7
**8b**	19.3 ± 5.6	20.5 ± 6.9
**8c**	10.7 ± 2.8	16.7 ± 7.0
**8d**	39.1 ± 4.2	51.0 ± 1.4
**8e**	8.7 ± 5.4	8.3 ± 5.5
**8f**	6.7 ± 3.6	12.2 ± 3.2
**8g**	12.9 ± 3.3	15.7 ± 4.9
**8h**	15.4 ± 4.9	19.0 ± 9.4
**8i**	18.1 ± 6.4	16.7 ± 5.6
**8j**	23.8 ± 5.2	22.9 ± 13.7
**8k**	5.7 ± 1.3	8.0 ± 4.3
**8l**	12.3 ± 6.1	12.4 ± 4.8
**8m**	13.6 ± 5.3	10.4 ± 2.8
**8n**	28.7 ± 10.5	32.7 ± 7.7
**8o**	27.0 ± 0.9	24.7 ± 1.0
**1a**	34.3 ± 2.2	48.3 ± 4.6
**2a**	0.484 ± 0.368	5.2 ± 1.4
**CA-4**	0.019 ± 0.012	3.1 ± 2.9
**Doxorubicin**	0.083 ± 0.021	0.274 ± 0.070
**Etoposide**	2.1 ± 0.4	6.3 ± 1.0

^a^ IC_50_ = compound concentration required to inhibit tumor cell proliferation by 50%. Data are expressed as the mean ± SE from the dose-response curves of at least three independent experiments with three determinations in each.

**Table 2:** Effect of **8f** and **8k** on cell cycle phase distribution of human U-937 leukemia cells.

Time	Compd	µM	% Sub-G_1_	% G_1_	% S	% G_2_-M
6 h		0	4.7 ± 0.2	47.3 ± 0.2	25.6 ± 0.1	20.7 ± 0.8
	**8f**	10	4.4 ± 0.5	40.4 ± 1.0 *	29.3 ± 0.1 *	23.6 ± 1.3
		30	3.8 ± 0.1	40.5 ± 1.6 *	30.9 ± 0.4 *	23.3 ± 1.4
	**8k**	10	3.7 ± 0.4	38.7 ± 0.3 *	30.7 ± 0.1 *	25.3 ± 0.2 *
		30	7.6 ± 0.5*	35.6 ± 0.5 *	32.0 ± 0.5 *	23.1 ± 0.4
						
12 h		0	2.3 ± 0.2	54.5 ± 0.6	21.4 ± 0.3	20.1 ± 0.3
	**8f**	10	6.5 ± 0.2 *	47.8 ± 1.0	22.4 ± 0.2	21.5 ± 1.2
		30	17.8 ± 1.0 *	27.4 ± 0.4 *	25.5 ± 0.1 *	27.6 ± 0.6 *
	**8k**	10	12.1 ± 0.8 *	41.9 ± 0.1 *	20.2 ± 0.4	23.7 ± 1.4
		30	24.3 ± 1.5 *	17.3 ± 0.4 *	26.8 ± 0.2 *	29.9 ± 1.7 *
						
24 h		0	3.4 ± 0.2	53.6 ± 0.2	24.5 ± 0.5	16.2 ± 0.5
	**8f**	10	10.3 ± 0.7 *	42.5 ± 0.3 *	29.8 ± 0.3 *	15.0 ± 0.8
		30	56.2 ± 2.0 *	12.6 ± 0.7 *	13.6 ± 0.6 *	16.6 ± 0.8
	**8k**	10	12.7 ± 0.1 *	39.4 ± 0.2 *	28.4 ± 1.1 *	17.2 ± 0.6
		30	51.5 ± 4.2 *	11.2 ± 0.9 *	11.5 ± 0.4 *	23.8 ± 2.9 *

Cells were cultured with the indicated concentrations of **8f** and **8k** for the indicated times, and the cell cycle phase distribution was determined by flow cytometry. The values are means ± SE of two independent experiments with three determinations in each. Asterisks indicate a significant difference (*p* < 0.05) compared with the corresponding controls.
